# Regulation of malonyl-CoA-acyl carrier protein transacylase network in umbilical cord blood affected by intrauterine hyperglycemia

**DOI:** 10.18632/oncotarget.20766

**Published:** 2017-09-08

**Authors:** Yong Zhang, Jianping Ye, Jianxia Fan

**Affiliations:** ^1^ International Peace Maternity and Child Health Hospital, School of Medicine, Shanghai Jiao Tong University, Shanghai 200030, China; ^2^ Institute of Embryo-Fetal Original Adult Disease Affiliated to Shanghai Jiao Tong University School of Medicine, Shanghai 200030, China; ^3^ Pennington Biomedical Research Center, Louisiana State University, Baton Rouge, LA 70808, USA

**Keywords:** pregnancy, MCAT, offspring, gestational diabetes, umbilical cord blood

## Abstract

**Background:**

Gestational diabetes mellitus (GDM) has been shown to be associated with high risk of diabetes in offspring. However, the mechanisms involved in the insulin resistance in offspring are still unclear. Mitochondrial dysfunction is related with insulin resistance. In mitochondria, malonyl-CoA-acyl carrier protein transacylase (MCAT) is the key enzyme of mitochondrial fatty acid synthesis and is estimated to contribute to insulin resistance. In this study, we aimed to examine the role of MCAT and its network in the umbilical cord blood in GDM-induced offspring insulin resistance.

**Methods:**

We isolated lymphocytes from umbilical cord vein blood in 6 GDM patients and 6 controls and examined the differences of RNA by RNA sequencing. qRT-PCR and western blot were used to measure mRNA and protein changes. Bisulfite genomic sequencing PCR was applied to detect DNA methylation.

**Results:**

We found more than 400 genes were differentially regulated in the lymphocytes of umbilical cord blood from GDM patients and these genes were mainly enriched in immune system and endocrine system, which relate to mitochondrial dysfunction and insulin resistance. MCAT closely related with PTPN1 (Protein Tyrosine Phosphatase, Non-Receptor Type1) and STAT5A (Signal Transducer And Activator of Transcription 5A), which were all increased in umbilical cord blood from GDM patients. Increase in MCAT may be due to decreased MCAT DNA methylation.

**Conclusion:**

MCAT and its network with PTPN1, STAT5A are regulated in umbilical cord blood affected by maternal intrauterine hyperglycemia.

## INTRODUCTION

It has been well documented that early-life environment plays an important role in adult health [1, 2]. Gestational diabetes mellitus (GDM), a common pregnancy complication, affects up to 28% of all pregnancies and contributes to the long term metabolic derangements both in mother and child [3, 4]. Perturbations in glucose and lipid metabolism may manifest early in children exposed to intrauterine hyperglycemia [5-7]. Although many studies have investigated the correlation between intrauterine hyperglycemia and the incidence of diabetes in offspring, the mechanisms of maternal intrauterine hyperglycemia induced offspring diabetes remain unclear.

Insulin resistance is the primary cause of type 2 diabetes and several factors have been proposed to explain the mechanisms of insulin resistance, such as lipotoxicity, inflammation, oxidative stress and mitochondrial dysfunction [8-10]. Mitochondrial dysfunction from pharmacological treatment or genetic manipulation has close relationship with insulin resistance [11-13]. Malonyl-CoA-acyl carrier protein transacylase (MCAT) is closely associated with FASII pathway of fatty acid biosynthesis in mitochondria [14]. The first committed step of fatty acid biosynthesis is the conversion of acetyl-CoA to malonyl-CoA by an ATP dependent acetyl-CoA carboxylase followed by the conversion of malonyl-CoA to malonyl-ACP through MCAT [15, 16]. MCAT overexpression can be used to improve fatty acid production by increasing the malonyl-ACP availability, and MCAT gene whole body knockout manifests primarily as a lipoylation defect, leading to decreased body weight, loss of white adipose tissue and energy disequilibrium [17]. However, specific roles of MCAT in insulin resistance are still unknown and need to be determined.

Herein we investigated the alteration of umbilical cord blood MCAT levels in offspring exposed to intrauterine hyperglycemia, and explored the network of MCAT in the mechanisms of insulin resistance. We hypothesized that MCAT expression in the umbilical cord blood from GDM patients would be increased, and the dramatically changed proteins in the network of MCAT may contribute to insulin resistance in the offspring.

## RESULTS

### Differential regulated genes by RNA sequencing in umbilical cord blood lymphocytes from normal pregnant women and GDM patients

**Figure 1:**
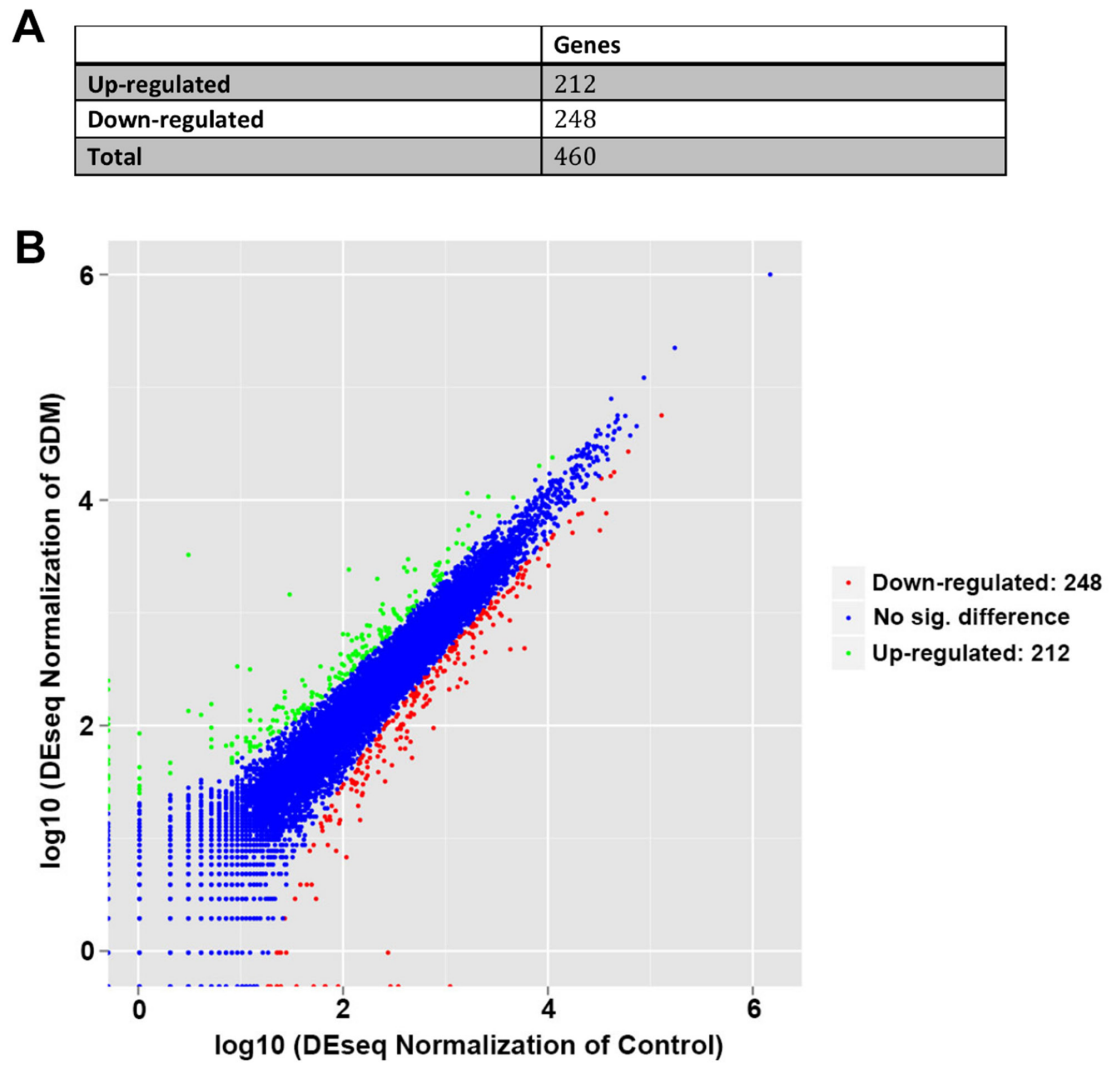
Differential regulated genes by RNA sequencing in umbilical cord blood lymphocytes from normal pregnant women and GDM patients **(A)** The number of identified genes. **(B)** Volcano plot presentation of the significantly altered gene profiles identified in umbilical cord blood lymphocytes. Up-regulated and down-regulated genes are indicated in dots of green and red, respectively.

By RNA sequencing, we found that there were 460 differentially expressed genes identified in the umbilical cord blood lymphocytes between normal pregnant women and GDM patients. Of those, 248 genes were down-regulated while 212 genes were up-regulated in the umbilical cord blood lymphocytes from GDM patients compared with those from normal pregnant women (Figure 1A, 1B and Supplementary Table 1).

### Gene ontology (GO) enrichment analysis of differential expressed genes

**Figure 2:**
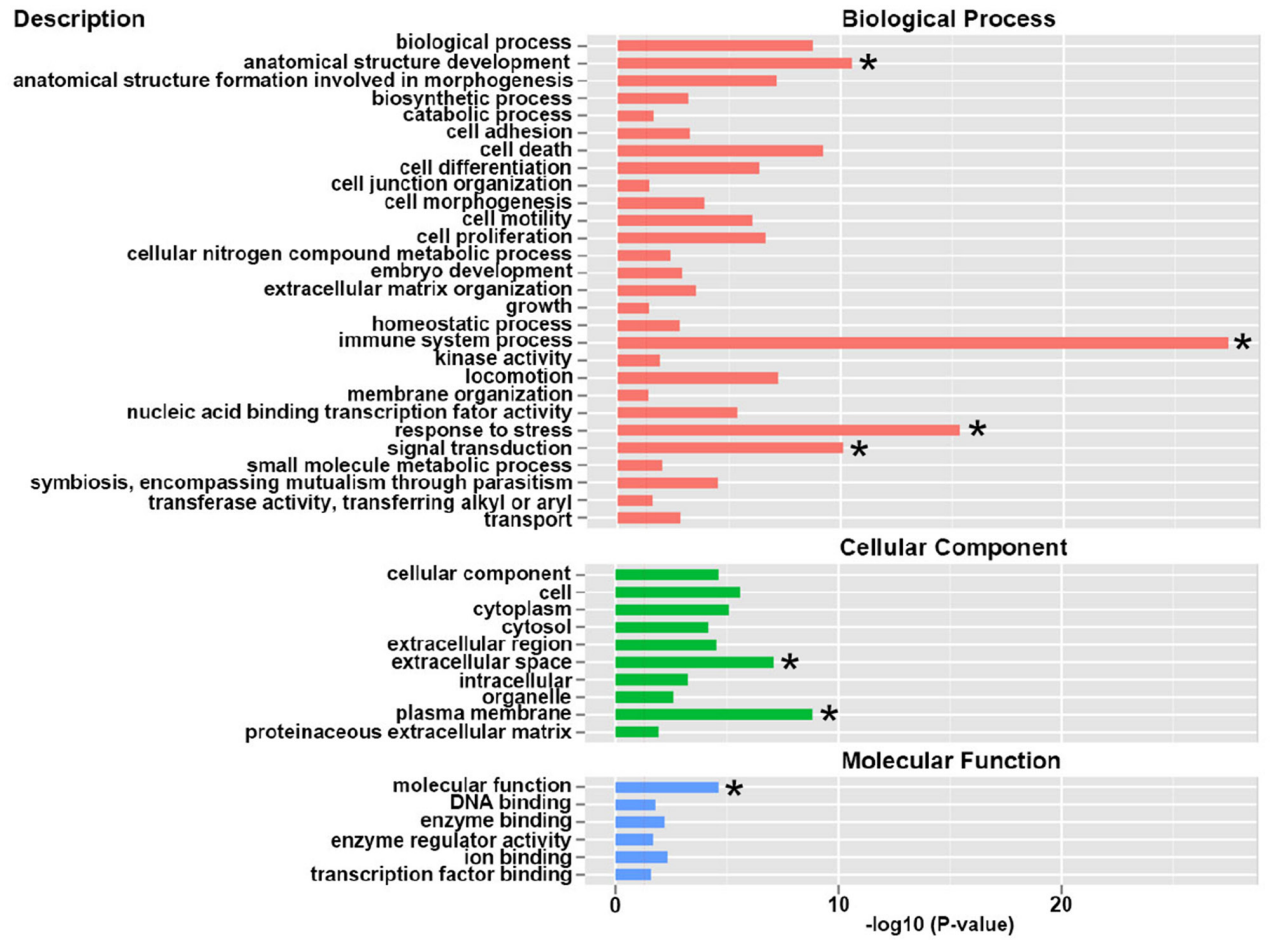
Gene Ontology (GO) enrichment analysis of differential expressed genes GO terms assigned to biological process, cellular component and molecular functions.

Through GO enrichment analysis, we found that these differential expressed genes were mainly involved in the biological process, cellular components and molecular function (Figure 2). In detail, the differential expressed genes were enriched in immune system process, response to stress and signal transduction. It is estimated that the genes involved in immune system, stress response and signal transduction were dramatically changed in the umbilical vein blood affected by intrauterine hyperglycemia.

### **Kyoto** encyclopedia of genes and genomes (KEGG) pathway classification enrichment analysis of differentiated expressed genes

**Figure 3:**
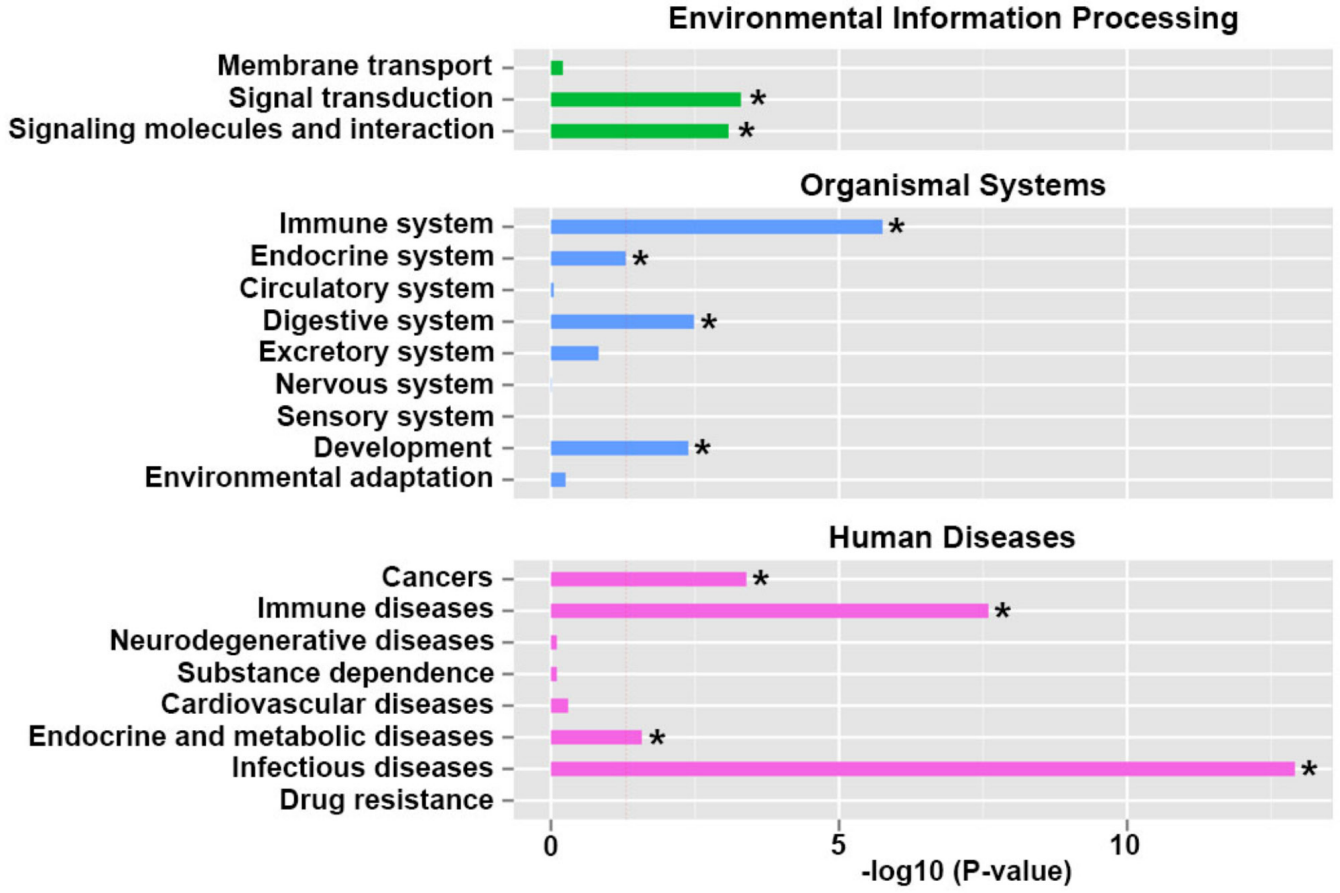
Kyoto Encyclopedia of Genes and Genomes (KEGG) pathway classification enrichment analysis of differentiated expressed genes Functional annotation and metabolic pathway analysis were grouped in environmental information processing, organismal systems and human diseases. The results are shown as the negative logarithm of significance, which is a statistical score and is a measure of the likelihood of the genes in a given network being found together as a result of chance, as determined by a Fisher’s exact test.

Through KEGG enrichment analysis, we checked the pathway classification enrichment of the differential regulated genes. In environmental information processing pathway, signal transduction and signaling molecules and interaction were dramatically regulated. In organismal systems pathways, immune system, endocrine system, digestive system and development pathways were significantly regulated. In human diseases pathways, cancers, immune diseases, endocrine and metabolic diseases and infectious diseases were dramatically affected (Figure 3). We assumed that the genes involved in endocrine system and its signaling pathways were significantly changed in the umbilical cord blood affected by intrauterine hyperglycemia.

### MCAT network by RNA sequencing

**Figure 4:**
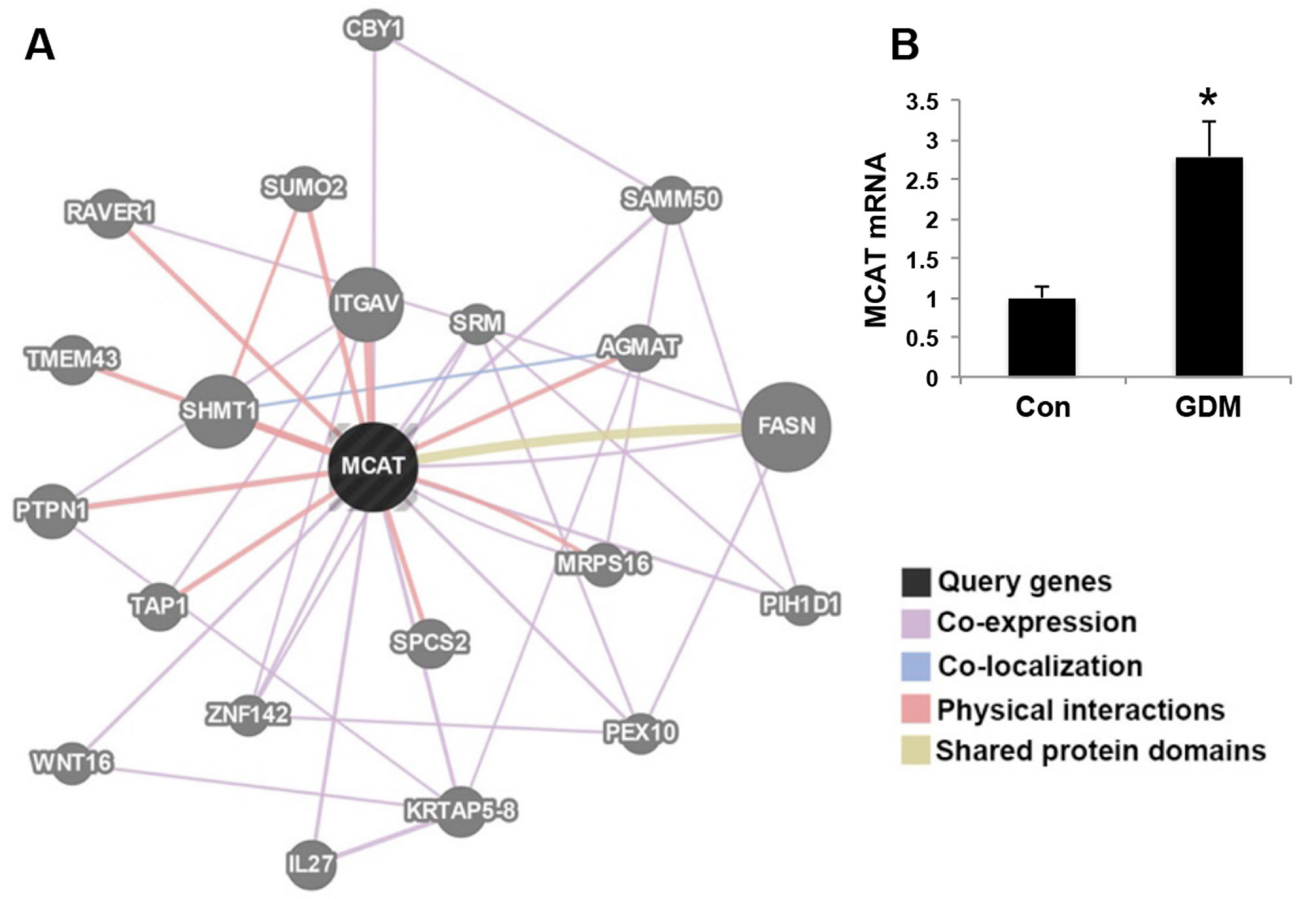
MCAT network by RNA sequencing **(A)** The query gene MCAT (marked black) has close relationship with the candidate genes (marked gray). The lines indicating the relationship of co-expression, co-localization, physical interactions and shared protein domains are marked pink, blue, red and yellow, respectively. **(B)** MCAT mRNA expression in umbilical cord blood from normal control pregnant women (Con) and GDM patients. N=6 in each group. *P<0.05 *vs.* Con.

From the RNA sequencing, we found out the network of MCAT (Figure 4A). MCAT gene expression was increased in the umbilical cord blood lymphocytes from GDM patients compared with that in normal pregnant women (Figure 4B). Many genes have the co-expression relationship with MCAT, such as KRTAP5-8, SAMM50, IL27, WNT16, ZNF142, PIH1D1, CBY1, SRM, PEX10, FASN, MRPS16. Some genes have physical interactions with MCAT, like ITGAV, SHMT1, PTPN1, SUMO2, TAP1, SPCS2, TMEM43, RAVER1, AGMAT and MRPS16. Besides, MCAT has shared protein domains with FASN (Table 1). This analysis was performed by GeneMANIA that find genes related to a set of input genes, using a very large set of functional association data. Association data include protein and genetic interactions, pathways, co-expression, co-localization and protein domain similarity.

**Table 1:** MCAT network associated genes

Gene 1	Gene 2	Network group	Networks
MCAT	PTPN1*	Physical interactions	Havugimana-Emili-2012
MCAT	STAT5A*	Co-expression	Wu-Garvey-2007
MCAT	KRTAP5-8	Co-expression	Innocenti-Brown-2011 Mallon-McKay-2013
MCAT	SAMM50	Co-expression	Burington-Shaughnessy-2008 Cheok-Evans-2003 Gysin-McMahon-2012 Salaverria-Siebert-2011
MCAT	IL27	Co-expression	Innocenti-Brown-2011 Mallon-McKay-2013
MCAT	WNT16	Co-expression	Gysin-McMahon-2012 Smirnov-Cheung-2009
MCAT	ZNF142	Co-expression	Burington-Shaughnessy-2008 Salaverria-Siebert-2011 Wu-Garvey-2007
MCAT	PIH1D1	Co-expression	Innocenti-Brown-2011 Kang-Willman-2010 Salaverria-Siebert-2011
MCAT	CBY1	Co-expression	Bild-Nevins-2006 B Gysin-McMahon-2012
MCAT	SRM	Co-expression	Roth-Zlotnik-2006 Smirnov-Cheung-2009 Wang-Maris-2006
MCAT	PEX10	Co-expression	Bild-Nevins-2006 B Burington-Shaughnessy-2008
MCAT	FASN	Co-expression	Bild-Nevins-2006 B
MCAT	MRPS16	Co-expression	Salaverria-Siebert-2011
MCAT	ITGAV	Physical interactions	Havugimana-Emili-2012
MCAT	SHMT1	Physical interactions	Havugimana-Emili-2012
MCAT	SUMO2	Physical interactions	BIOGRID-SMALL-SCALE-STUDIES
MCAT	TAP1	Physical interactions	Havugimana-Emili-2012
MCAT	SPCS2	Physical interactions	Havugimana-Emili-2012
MCAT	TMEM43	Physical interactions	Havugimana-Emili-2012
MCAT	RAVER1	Physical interactions	Havugimana-Emili-2012
MCAT	AGMAT	Physical interactions	Havugimana-Emili-2012
MCAT	MRPS16	Physical interactions	Havugimana-Emili-2012
MCAT	FASN	Shared protein domains	INTERPRO PFAM

MCAT: Malony-CoA-Acyl Carrier Protein Transacylase, PTPN1: Protein Tyrosine Phosphatase, Non-Receptor Type 1, STAT5A: Signal Transducer And Activator Of Transcription 5A, KRTAP5-8: Keratin Associated Protein 5-8, SAMM50: Sorting And Assembly Machinery Component 50, IL27: Interleukin 27, WNT16: Wnt Family Member 16, ZNF142: Zinc Finger Protein 142, PIH1D1: PIH1 Domain Containing 1, CBY1: Chibby Homolog 1, SRM: Spermidine Synthase, PEX10: Peroxisomal Biogenesis Factor 10, FASN: Fatty Acid Synthase, MRPS16: Mitochondrial Ribosomal Protein S16, ITGAV: Integrin Subunit Alpha V, SHMT1: Serine Hydroxymethyltransferase 1, SUMO2: Small Ubiquitin-Like Modifier 2, TAP1: Transporter 1, SPCS2: Signal Peptidase Complex Subunit 2, TMEM43: Transmembrane Protein 43, RAVER1: Ribonucleoprotein, AGMAT: Agmatinase.

We found that some genes like PTPN1 (Protein Tyrosine Phosphatase, Non-Receptor Type1) and STAT5A (Signal Transducer And Activator of Transcription 5A), which were involved in insulin signaling pathways had close relationship with MCAT (Table 1), it is possible that the pathways of MCAT may be novel mechanisms as to the offspring insulin resistance affected by intrauterine hyperglycemia.

### MCAT, PTPN1 and STAT5A expression in lymphocytes of umbilical cord blood from normal pregnant women and GDM patients

**Figure 5:**
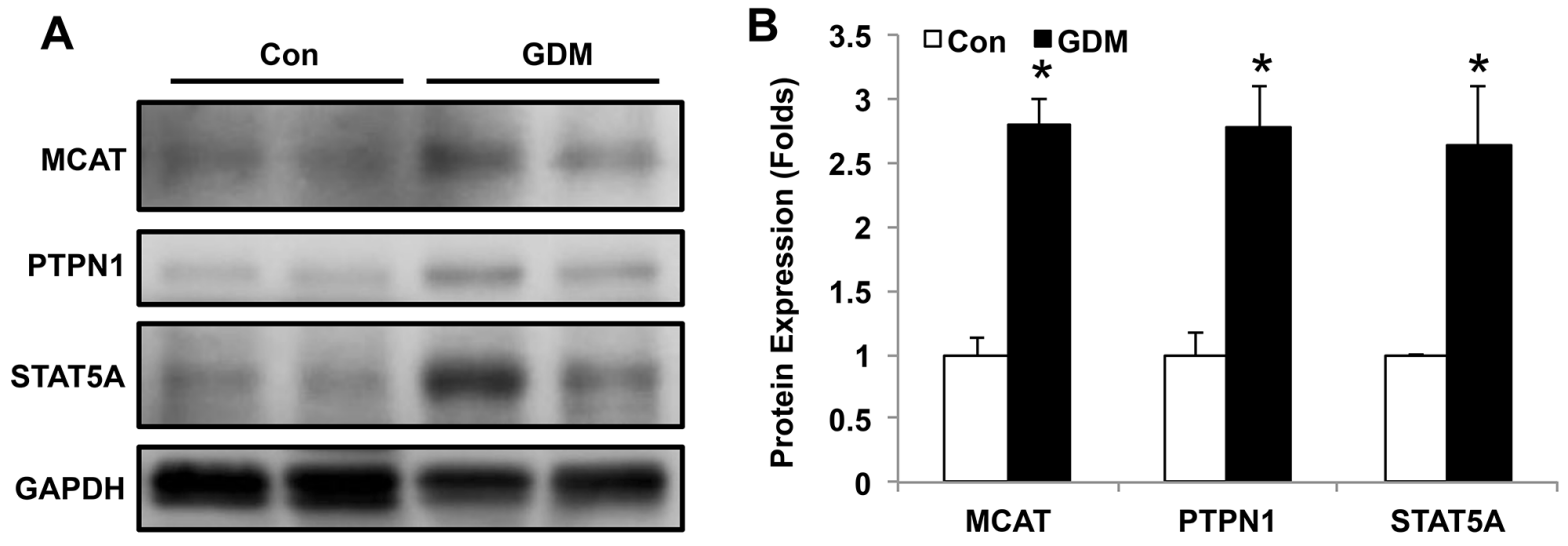
MCAT, PTPN1 and STAT5A expression in lymphocytes of umbilical cord blood from normal pregnant women and GDM patients **(A)** Representative western blot of MCAT, PTPN1 and STAT5A protein expression in umbilical cord blood from normal control pregnant women (Con) and GDM patients. **(B)** Bar figure of the statistical analysis for MCAT, PTPN1 and STAT5A protein quantification. N=6 in each group. * *P*<0.05 *vs.* Con.

We further verified the MCAT network by Western blotting in umbilical cord blood from both normal control pregnant women and GDM patients. As shown in Figure 5A and Supplementary Figure 1, the protein expressions of MCAT, PTPN1 and STAT5A were increased in the lymphocytes of umbilical cord blood from GDM patients compared with those in healthy pregnant women, with significant differences by quantification (Figure 5B). These data suggest that the signaling pathways that involved MCAT, PTPN1 and STAT5A may contribute to offspring insulin resistance affected by intrauterine hyperglycemia.

### MCAT methylation in umbilical cord blood lymphocytes from normal pregnant women and GDM patients

**Figure 6:**
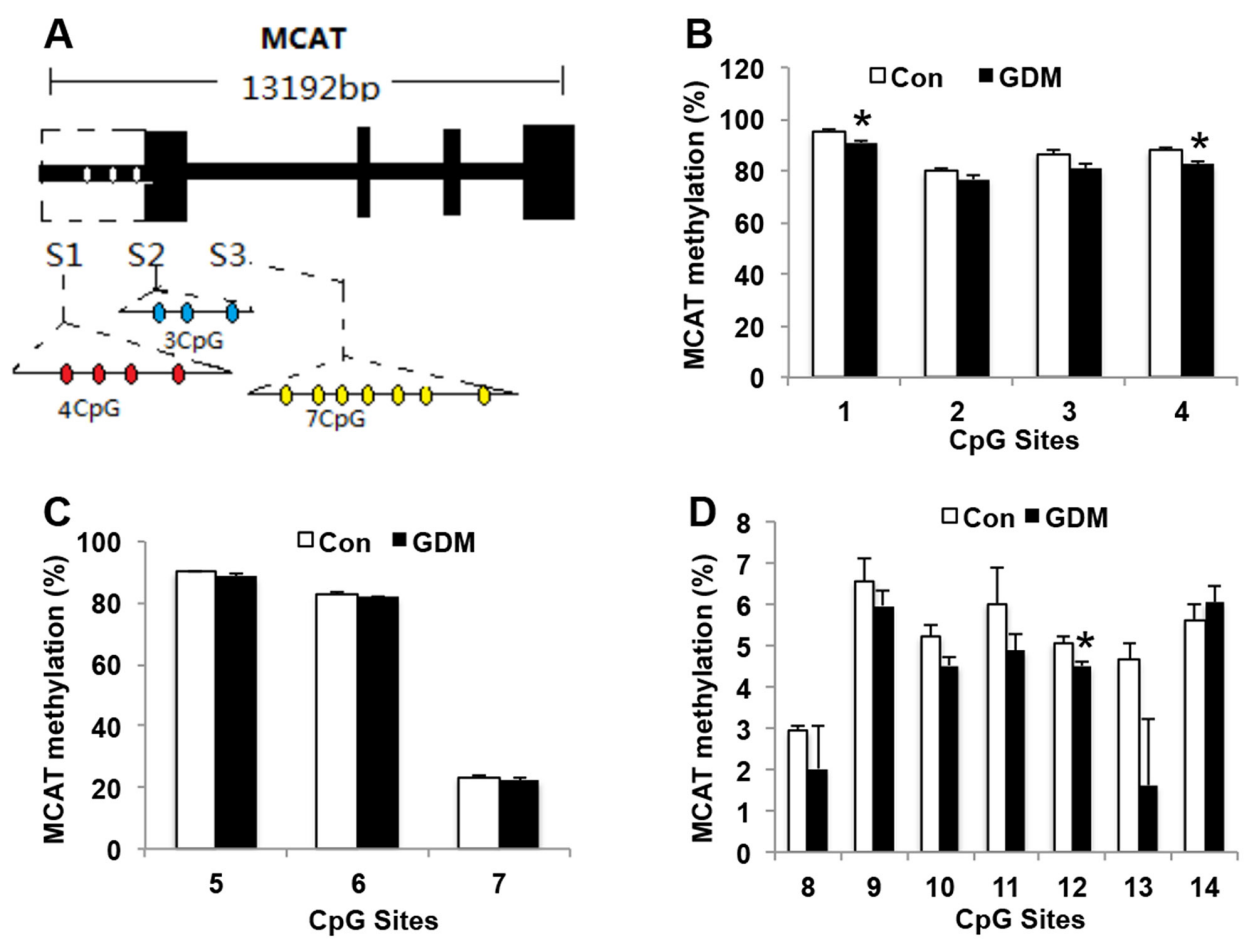
MCAT methylation in umbilical cord blood lymphocytes from normal pregnant women and GDM patients **(A)** Schematic diagram of the cytosine phosphate guanine (CpG) sites for MCAT promoter. **(B)** Methylation levels in CpG sites 1 to 4 in *MCAT* DMR1 (S1). **(C)** Methylation levels in CpG sites 5 to 7 in *MCAT* DMR2 (S2). **(D)** Methylation levels in CpG sites 8 to 14 in *MCAT* DMR3 (S3). N=3 in each group. * *P*<0.05 *vs.* Con.

MCAT allelic expression is regulated by allele-specific methylation at 3 differentially methylated regions (DMRs). We collected the lymphocytes of umbilical cord blood from 3 control pregnant women and 3 GDM patients, and analyzed the methylation levels of 4 cytosine phosphate guanine (CpGs) of the *MCAT* DMR1, 3 CpGs of the *MCAT* DMR2, and 7 CpGs of the *MCAT* DMR3 by bisulfite genomic sequencing PCR (Figure 6A). We found that in *MCAT* DMR1, the methylation status of CpG sites 1 and 4 was hypomethylated in GDM patients (90.85 ± 0.76% for site 1 and 82.33 ± 1.37% for site 4, respectively) compared with control (95.24 ± 0.93% for site 1 and 87.78 ± 1.15% for site 4, respectively) (Figure 6B). In *MCAT* DMR2, the methylation status in control and GDM groups did not show significant differences (Figure 6C). In *MCAT* DMR3, CpG site 12 showed less methylation in GDM group (4.51 ± 0.10%) than control group (5.06 ± 0.14%) (Figure 6D). The decreased methylation status of MCAT in GDM groups may account for the increased gene expression of MCAT in the lymphocytes of umbilical cord blood from GDM patients compared with the control group.

## DISCUSSION

In the present study, we found that MCAT, PTPN1 and STAT5A were closely associated and all increased in the lymphocytes of umbilical cord blood from GDM patients compared with those in healthy pregnant women. Since PTPN1 and STAT5A were demonstrated to impair insulin signaling, MCAT and its network may be novel mechanisms as to the offspring insulin resistance affected by intrauterine hyperglycemia.

This study proposes for the first time that MCAT network contributes to the offspring insulin resistance from GDM mothers. MCAT mRNA and protein were elevated in the lymphocytes of umbilical cord blood from GDM mothers. MCAT DNA methylation was decreased in several CpG sites of MCAT promoter, which may account for the increased MCAT mRNA level. Since MCAT is of vital importance in fatty acid synthesis, the increased ability of fatty acid synthesis in GDM mothers can pass to the offspring through the lymphocytes of umbilical cord blood. The specific role of MCAT in insulin resistance has not been determined. It is estimated that increased MCAT expression will lead to enhanced fatty acid synthesis and lipotoxicity, which may induce insulin resistance. It has been demonstrated that MCAT whole body knockout (KO) mice showed compromised mitochondrial fatty acid synthesis leading to loss of white adipose tissue and disruption of energy metabolism [17]. The specific role of liver or adipose tissue MCAT in insulin resistance needs to be explored in future study.

Through MCAT net analysis, we found that MCAT can physically interact with PTPN1 and co-expressed with STAT5A. PTPN1 has been shown to act as a negative regulator of insulin signaling by dephosphorylating the phosphotyrosine residues of insulin receptor kinase [18, 19]. Loss of PTPN1 activity causes enhanced insulin sensitivity and resistance to weight gain in mice [20]. Further, liver specific PTPN1 deficiency improves hepatic insulin sensitivity and whole body glucose homeostasis [21, 22]. Therefore, inhibition of PTPN1 has been proposed to be a potential therapy for obesity, insulin resistance and type-2 diabetes mellitus [23]. Besides, STAT5 activity in pancreatic beta cells influences the severity of diabetes in animal models of type 1 and 2 diabetes [24]. The STAT5A-mediated induction of pyruvate dehydrogenase kinase 4 expression by prolactin results in an inhibition of insulin-stimulated glucose transport in fat cells and contributes to insulin resistance [25]. Furthermore, intrauterine hyperglycemia induced offspring glucose intolerance by inhibiting pyruvate dehydrogenase (PDH) activity, along with increased PDH phosphorylation in the liver [26]. The increased PTPN1 and STAT5A in the lymphocytes of umbilical core blood in GDM mothers are proposed to contribute to offspring insulin resistance. Besides PTPN1 and STAT5A, some other genes like FASN, TAP1, which are also involved in insulin resistance [27, 28], have close relationship with MCAT. The potential relationship between MCAT and these genes will be explored in the future study.

In conclusion, MCAT and its crosstalk with PTPN1, STAT5A are increased in the umbilical cord blood affected by maternal uterine hyperglycemia. Inhibition of MCAT by pharmacological treatment in clinic may be a novel way to cure diabetes.

## MATERIALS AND METHODS

### Isolation of lymphocytes from umbilical cord blood

The umbilical cord blood was collected from both normal pregnant women and GDM pregnant women after delivering babies in International Peace Maternity and Child Hospital and was approved by Shanghai Jiaotong University Ethics Committee. Diagnosis for GDM: fasting blood glucose level greater than 5.1 mmol/l or 1 and 2 h blood glucose levels after the oral glucose tolerance test (OGTT) greater than 10.0 mmol/l and 8.5 mmol/l, respectively. OGTT was conducted within 24^th^-28^th^ weeks of pregnancy. The blood was diluted with sterile PBS and poured carefully onto the Ficoll solution. The tubes were centrifuged at 400 *g* for 20 min and the ring with lymphocytes were harvested without touching the Ficoll. The lymphocytes were then washed twice with PBS and used for further study.

### Extraction of total RNA

Total RNA was extracted using RNAiso Plus Total RNA extraction reagent (TaKaRa, Shiga, Japan) following the manufacturer’s instructions. The residual genomic DNA in total RNA was removed by DNase I (MBI Fermentas, Glen Burnie, MD, USA). Total RNA was dissolved in RNase-free water and RNA integrity was measured using Agilent 2100 bioanalyzer (Quantifluor-ST fluorometer, Promega, E6090). The high quality RNA was used for cDNA library construction and Illumina sequencing.

### Sequencing

Poly (A) mRNA was isolated from the total RNA using the PolyA+ Tract mRNA Isolation System (Illumina, San Diego, CA), and further purified using the RNeasy MinElute Clean up Kit (Qiagen, Hilden, Germany) following the manufacturer’s protocol. Fragmentation buffer was added to cleave mRNA into short fragments, and these fragments were used to synthesize first-strand cDNA using random hexamer primers, which was further transformed into double stranded cDNA with RHase H and DNA polymerase I. A paired-end library was constructed from the cDNA synthesized using the Genomic Sample Prep Kit (Illumina). Fragments larger than 375 bp were purified with QIAquick PCR Extraction Kit (Qiagen), end-repaired, and linked with sequencing adapters. AMPureXP beads were used to remove the unsuitable fragments, and the sequencing library was constructed with PCR amplification. After being validated using Pico green staining (Quant-iT PicoGreen dsDNA Assay Kit, Invitrogen, P7589) and fluorospectrophotometry, and quantified using Agilent 2100 (Quantifluor-ST fluorometer, Promega, E6090), the library was sequenced using Illumina Miseq Platform (Shanghai Personal Biotechnology Cp., Ltd. Shanghai, China). The read length was 2×150 bp, and 6 Gbp sequencing runs were performed. For subsequent analysis, 1/2 run data was generated.

### Quantitative real-time RT-PCR (qRT-PCR)

SYBR Green RT-PCR reaction was used to quantify mRNA. The total RNA was prepared from tissues or cells with TRIzol reagent (Sigma, St. Louis, MO). The assay was conducted with 7900 HT Fast real-time PCR System (Applied Biosystems, Foster City, CA). The target mRNA signal was normalized with GAPDH RNA.

### DNA methylation (bisulfite genomic sequencing PCR)

Genomic DNA was extracted from lymphocytes of umbilical cord blood in control and GDM groups. Bisulfite was converted using the EpiTect bisulfite kit (Qiagen, Valencia, CA) to deaminate cytosine to uracil according to the manufacturer’s instructions; 5-methyl-cytosine was protected from deamination. Analysis of the methylation status of the *MCAT* DMR was determined by cloning and sequencing of bisulfite-treated DNA. The purified PCR products were cloned using the pMD19-T vector system (TaKaRa, Dalian, China). The sequence obtained by cloning was analyzed with 3730 DNA Analyzer polymers (Applied Biosystems, Carlsbad, CA).

### Western blot analysis

Cell preparations were sonicated in lysis buffer and 50 μg protein was resolved on a 8% SDS-PAGE gel and electroblotted onto PVDF membrane. Blots were then blocked in 5% nonfat milk containing 0.05% Tween-20, rinsed in PBS (pH 7.4), and incubated with the following antibodies: mouse monoclonal anti-MCAT (1:1000), rabbit monoclonal anti-PTPN1 (1:1000), rabbit monoclonal anti-STAT5A (1:1000) and rabbit polyclonal anti-GAPDH (1:1000). GAPDH was probed as a loading control. The immunoreactivity was visualized by enhanced chemiluminescence substrate system (ECL). Films were scanned and subsequently analyzed by measuring the optical densities of immunostained bands using an image-processing and analysis system (Bio-Rad, Hercules, CA).

### Statistical analysis

The tool used for the differential expression analysis was DESeq, which is an R package to analyze count data from high-throughput sequencing assays such as RNA-Seq and test for differential expression. We used the software named MGI GO Term Finder to perform GO enrichment analysis, which was established to provide a common language to describe aspects of a gene product’s biology. The method of KEGG enrichment analysis was similar to GO enrichment analysis and performed the same arithmetic. We detected MCAT gene promoter region methylation pattern using pyrosequencing.

In this study, the data were presented as the mean ± SEM from multiple samples and each experiment was conducted at least three times. The representative immunoblot is presented. In the statistical analysis, two-tailed, unpaired Student’s t test was used in analysis of the data with significance *P*< 0.05.

## SUPPLEMENTARY MATERIALS FIGURE AND TABLE





## Abbreviations

MCAT: Malonyl-CoA-Acyl Carrier Protein Transacylase
PDH: Pyruvate Dehydrogenase
GDM: Gestational Diabetes Mellitus
GO: Gene Ontology
KEGG: Kyoto Encyclopedia of Genes and Genomes
DMR: Differentially Methylated Region
CpG: Cytosine phosphate Guanine
PTPN1: Protein Tyrosine Phosphatase Non-Receptor Type 1
STAT5A: Signal Transducer And Activator Of Transcription 5A
KRTAP5-8: Keratin Associated Protein 5-8
SAMM50: Sorting And Assembly Machinery Component 50
IL27: Interleukin 27
WNT16: Wnt Family Member 16
ZNF142: Zinc Finger Protein 142
PIH1D1: PIH1 Domain Containing 1
CBY1: Chibby Homolog 1
SRM: Spermidine Synthase
PEX10: Peroxisomal Biogenesis Factor 10
FASN: Fatty Acid Synthase
MRPS16: Mitochondrial Ribosomal Protein S16
ITGAV: Integrin Subunit Alpha V
SHMT1: Serine Hydroxymethyltransferase 1
SUMO2: Small Ubiquitin-Like Modifier 2
TAP1: Transporter 1
SPCS2: Signal Peptidase Complex Subunit 2
TMEM43: Transmembrane Protein 43
RAVER1: Ribonucleoprotein
AGMAT: Agmatinase
