# Supplementary material for: Regulation of malonyl-CoA-acyl carrier protein transacylase network in umbilical cord blood affected by intrauterine hyperglycemia
Source: Oncotarget. 2017 Sep 8;8(43):75254–63. doi: 10.18632/oncotarget.20766 (PMC5650417; doi:10.18632/oncotarget.20766)
Supplement: Supplementary file 1 [file oncotarget-08-75254-s001.pdf]

Regulation of malonyl-CoA-acyl carrier protein transacylase network in umbilical cord blood affected by intrauterine hyperglycemia

SUPPLEMENTARY MATERIALS

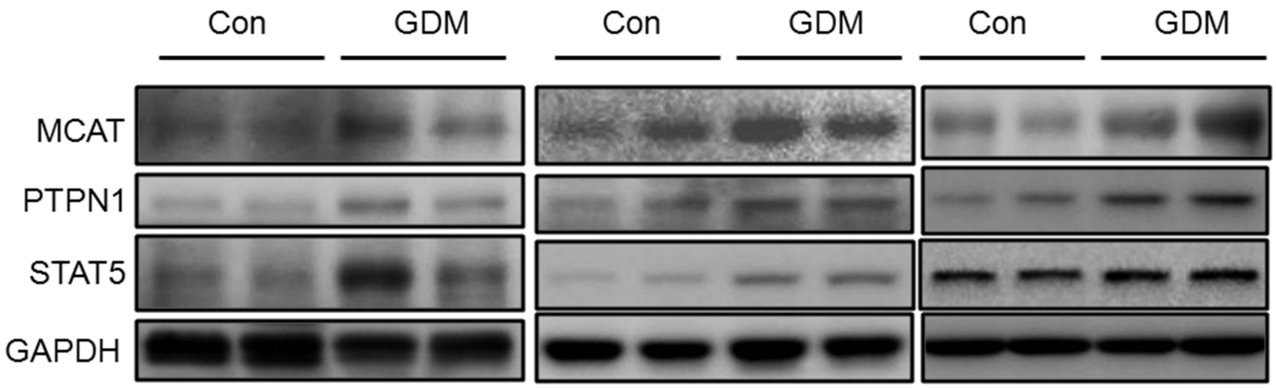

Supplementary Figure 1:MCAT, PTPN1 and STAT5A expression in lymphocytes of umbilical cord blood from 6 normal pregnant women and 6 GDM patients.

Supplementary Table 1: Top 100 under and over expressed genes in umbilical vein blood lymphocytes from GDM patients by RNA sequencing.

See Supplementary File 1
