# Supplementary material for: Regulation of malonyl-CoA-acyl carrier protein transacylase network in umbilical cord blood affected by intrauterine hyperglycemia
Source: Oncotarget. 2017 Sep 8;8(43):75254–63. doi: 10.18632/oncotarget.20766 (PMC5650417; doi:10.18632/oncotarget.20766)
Supplement: Supplementary file 2 [file oncotarget-08-75254-s002.docx]

**Supplementary Table 1: Top 100 under and over expressed genes in umbilical vein blood lymphocytes from GDM patients by RNA sequencing**

| **Genes** | **Con** | **GDM** | **GDM vs. Con** | |
| --- | --- | --- | --- | --- |
| **Top 100 under-expressed** | **Mean** | **Mean** | **Ratio** | **p value** |
| HLA class II histocompatibility antigen, DR beta 3chain [Source:UniProtKB/Swiss-Prot;Acc:P79483] | 2.75E+02 | 9.69E-01 | 0.004 | 0.000 |
| ATPase, Na+/K+ transporting, alpha 4 polypeptide[Source:HGNC Symbol;Acc:HGNC:14073] | 2.79E+01 | 9.69E-01 | 0.035 | 0.021 |
| ets variant 7 [Source:HGNC Symbol;Acc:HGNC:18160]" | 2.48E+01 | 9.69E-01 | 0.039 | 0.034 |
| UDP-GlcNAc:betaGal beta-1,3-N-acetylglucosaminyltransferase-like 1 [Source:HGNCSymbol;Acc:HGNC:21727] | 2.37E+01 | 9.69E-01 | 0.041 | 0.040 |
| hydroxycarboxylic acid receptor 3 [Source:HGNCSymbol;Acc:HGNC:16824] | 2.27E+01 | 9.69E-01 | 0.043 | 0.046 |
| leukocyte immunoglobulin-like receptor, subfamily A(with TM domain), member 4 [Source:HGNCSymbol;Acc:HGNC:15503] | 2.27E+01 | 9.69E-01 | 0.043 | 0.046 |
| carboxylesterase 1 [Source:HGNCSymbol;Acc:HGNC:1863] | 5.47E+01 | 2.91E+00 | 0.053 | 0.003 |
| solute carrier family 6 (neutral amino acidtransporter), member 19 [Source:HGNCSymbol;Acc:HGNC:27960] | 1.07E+02 | 6.78E+00 | 0.063 | 0.000 |
| solute carrier family 2 (facilitated glucosetransporter), member 4 [Source:HGNCSymbol;Acc:HGNC:11009] | 2.68E+01 | 1.94E+00 | 0.072 | 0.046 |
| major histocompatibility complex, class II, DQalpha 2 [Source:HGNC Symbol;Acc:HGNC:4943] | 4.95E+01 | 3.88E+00 | 0.078 | 0.008 |
| early growth response 1 [Source:HGNCSymbol;Acc:HGNC:3238] | 5.92E+03 | 4.84E+02 | 0.082 | 0.000 |
| carboxypeptidase A5 [Source:HGNCSymbol;Acc:HGNC:15722] | 3.40E+01 | 2.91E+00 | 0.085 | 0.030 |
| C-type lectin domain family 4, member F[Source:HGNC Symbol;Acc:HGNC:25357] | 4.44E+01 | 3.88E+00 | 0.087 | 0.014 |
| frizzled class receptor 2 [Source:HGNCSymbol;Acc:HGNC:4040] | 8.56E+01 | 7.75E+00 | 0.091 | 0.001 |
| NK6 homeobox 3 [Source:HGNC Symbol;Acc:HGNC:26328]" | 1.47E+02 | 1.45E+01 | 0.099 | 0.000 |
| UBX domain protein 10 [Source:HGNCSymbol;Acc:HGNC:26354] | 3.82E+01 | 3.88E+00 | 0.102 | 0.028 |
| tripartite motif containing 69 [Source:HGNCSymbol;Acc:HGNC:17857] | 3.82E+01 | 3.88E+00 | 0.102 | 0.028 |
| espin [Source:HGNC Symbol;Acc:HGNC:13281]" | 4.73E+02 | 5.14E+01 | 0.109 | 0.000 |
| ras homolog family member B [Source:HGNCSymbol;Acc:HGNC:668] | 4.28E+03 | 4.72E+02 | 0.110 | 0.000 |
| lipocalin 8 [Source:HGNC Symbol;Acc:HGNC:27038]" | 7.12E+01 | 8.72E+00 | 0.123 | 0.007 |
| glutathione S-transferase mu 4 [Source:HGNCSymbol;Acc:HGNC:4636] | 2.57E+02 | 3.20E+01 | 0.124 | 0.000 |
| chemokine (C-X3-C motif) receptor 1 [Source:HGNCSymbol;Acc:HGNC:2558] | 7.62E+02 | 9.50E+01 | 0.125 | 0.000 |
| interferon-induced protein with tetratricopeptiderepeats 1 [Source:HGNC Symbol;Acc:HGNC:5407] | 1.40E+02 | 1.94E+01 | 0.138 | 0.001 |
| kinesin family member 26A [Source:HGNCSymbol;Acc:HGNC:20226] | 9.18E+01 | 1.36E+01 | 0.148 | 0.006 |
| yippee-like 4 (Drosophila) [Source:HGNCSymbol;Acc:HGNC:18328] | 3.00E+02 | 4.46E+01 | 0.148 | 0.000 |
| transmembrane protein 176A [Source:HGNCSymbol;Acc:HGNC:24930] | 1.59E+03 | 2.40E+02 | 0.151 | 0.000 |
| v-myc avian myelocytomatosis viral oncogene lungcarcinoma derived homolog [Source:HGNCSymbol;Acc:HGNC:7555] | 4.02E+02 | 6.20E+01 | 0.154 | 0.000 |
| neuregulin 1 [Source:HGNC Symbol;Acc:HGNC:7997]" | 9.39E+01 | 1.45E+01 | 0.155 | 0.006 |
| suppressor APC domain containing 2 [Source:HGNCSymbol;Acc:HGNC:28055] | 1.54E+02 | 2.42E+01 | 0.158 | 0.002 |
| zinc finger protein 703 [Source:HGNCSymbol;Acc:HGNC:25883] | 4.55E+02 | 7.17E+01 | 0.158 | 0.000 |
| beta-1,4-N-acetyl-galactosaminyl transferase 3[Source:HGNC Symbol;Acc:HGNC:24137] | 1.55E+02 | 2.52E+01 | 0.163 | 0.002 |
| glutathione S-transferase mu 5 [Source:HGNCSymbol;Acc:HGNC:4637] | 4.75E+01 | 7.75E+00 | 0.163 | 0.039 |
| FBJ murine osteosarcoma viral oncogene homolog[Source:HGNC Symbol;Acc:HGNC:3796] | 3.22E+04 | 5.39E+03 | 0.167 | 0.000 |
| sialic acid binding Ig-like lectin 1, sialoadhesin[Source:HGNC Symbol;Acc:HGNC:11127] | 1.30E+03 | 2.20E+02 | 0.169 | 0.000 |
| otoferlin [Source:HGNC Symbol;Acc:HGNC:8515]" | 5.16E+01 | 8.72E+00 | 0.169 | 0.035 |
| chemokine (C-C motif) receptor 2 [Source:HGNCSymbol;Acc:HGNC:1603] | 3.60E+02 | 6.20E+01 | 0.172 | 0.000 |
| solute carrier family 6 (neurotransmittertransporter, glycine), member 9 [Source:HGNCSymbol;Acc:HGNC:11056] | 2.21E+02 | 3.88E+01 | 0.176 | 0.001 |
| family with sequence similarity 118, member A[Source:HGNC Symbol;Acc:HGNC:1313] | 2.45E+03 | 4.46E+02 | 0.182 | 0.000 |
| MX dynamin-like GTPase 1 [Source:HGNCSymbol;Acc:HGNC:7532] | 1.40E+03 | 2.58E+02 | 0.185 | 0.000 |
| leukocyte immunoglobulin-like receptor, subfamily B(with TM and ITIM domains), member 5 [Source:HGNCSymbol;Acc:HGNC:6609] | 6.29E+01 | 1.16E+01 | 0.185 | 0.028 |
| Rh blood group, D antigen [Source:HGNCSymbol;Acc:HGNC:10009] | 4.17E+02 | 7.75E+01 | 0.186 | 0.000 |
| 2'-5'-oligoadenylate synthetase 1, 40/46kDa[Source:HGNC Symbol;Acc:HGNC:8086] | 1.09E+03 | 2.08E+02 | 0.191 | 0.000 |
| kinase non-catalytic C-lobe domain (KIND)containing 1 [Source:HGNC Symbol;Acc:HGNC:29374] | 1.57E+02 | 3.00E+01 | 0.192 | 0.004 |
| adenosine deaminase, RNA-specific, B2 (non-functional) [Source:HGNC Symbol;Acc:HGNC:227] | 1.36E+02 | 2.62E+01 | 0.192 | 0.005 |
| tumor necrosis factor receptor superfamily, member8 [Source:HGNC Symbol;Acc:HGNC:11923] | 2.52E+02 | 4.85E+01 | 0.192 | 0.001 |
| coiled-coil alpha-helical rod protein 1[Source:HGNC Symbol;Acc:HGNC:13930] | 7.53E+01 | 1.45E+01 | 0.193 | 0.021 |
| transmembrane protein 176B [Source:HGNCSymbol;Acc:HGNC:29596] | 4.27E+03 | 8.58E+02 | 0.201 | 0.000 |
| cerebellin 3 precursor [Source:HGNCSymbol;Acc:HGNC:20146] | 1.68E+02 | 3.39E+01 | 0.202 | 0.004 |
| transmembrane protein 60 [Source:HGNCSymbol;Acc:HGNC:21754] | 6.19E+01 | 1.26E+01 | 0.204 | 0.037 |
| dual specificity phosphatase 1 [Source:HGNCSymbol;Acc:HGNC:3064] | 3.72E+04 | 7.60E+03 | 0.204 | 0.000 |
| olfactomedin 1 [Source:HGNC Symbol;Acc:HGNC:17187]" | 8.46E+01 | 1.74E+01 | 0.206 | 0.020 |
| protease, serine, 21 (testisin) [Source:HGNCSymbol;Acc:HGNC:9485] | 1.72E+02 | 3.59E+01 | 0.208 | 0.004 |
| translocator protein 2 [Source:HGNCSymbol;Acc:HGNC:21256] | 4.23E+02 | 8.82E+01 | 0.208 | 0.001 |
| interferon, alpha-inducible protein 27 [Source:HGNCSymbol;Acc:HGNC:5397] | 7.43E+01 | 1.55E+01 | 0.209 | 0.027 |
| EPH receptor B2 [Source:HGNC Symbol;Acc:HGNC:3393]" | 6.91E+01 | 1.45E+01 | 0.210 | 0.032 |
| neurotensin receptor 1 (high affinity) [Source:HGNCSymbol;Acc:HGNC:8039] | 3.65E+02 | 7.85E+01 | 0.215 | 0.001 |
| interferon, alpha-inducible protein 6 [Source:HGNCSymbol;Acc:HGNC:4054] | 1.85E+03 | 4.03E+02 | 0.218 | 0.000 |
| atonal homolog 8 (Drosophila) [Source:HGNCSymbol;Acc:HGNC:24126] | 6.09E+01 | 1.36E+01 | 0.223 | 0.048 |
| atypical chemokine receptor 1 (Duffy blood group)[Source:HGNC Symbol;Acc:HGNC:4035] | 1.21E+02 | 2.71E+01 | 0.225 | 0.013 |
| carbohydrate (chondroitin 4) sulfotransferase 13[Source:HGNC Symbol;Acc:HGNC:21755] | 3.29E+02 | 7.46E+01 | 0.227 | 0.002 |
| interferon-induced protein with tetratricopeptiderepeats 2 [Source:HGNC Symbol;Acc:HGNC:5409] | 1.36E+02 | 3.10E+01 | 0.228 | 0.011 |
| CDC42 effector protein (Rho GTPase binding) 2[Source:HGNC Symbol;Acc:HGNC:16263] | 1.31E+02 | 3.00E+01 | 0.229 | 0.012 |
| cytochrome P450, family 27, subfamily A,polypeptide 1 [Source:HGNC Symbol;Acc:HGNC:2605] | 1.53E+03 | 3.52E+02 | 0.230 | 0.000 |
| 1-acylglycerol-3-phosphate O-acyltransferase 1[Source:HGNC Symbol;Acc:HGNC:324] | 6.60E+01 | 1.55E+01 | 0.235 | 0.047 |
| G protein-coupled bile acid receptor 1 [Source:HGNCSymbol;Acc:HGNC:19680] | 7.03E+02 | 1.68E+02 | 0.239 | 0.001 |
| prostaglandin-endoperoxide synthase 2(prostaglandin G/H synthase and cyclooxygenase)[Source:HGNC Symbol;Acc:HGNC:9605] | 6.57E+02 | 1.57E+02 | 0.239 | 0.001 |
| methyltransferase like 7A [Source:HGNCSymbol;Acc:HGNC:24550] | 6.45E+02 | 1.55E+02 | 0.240 | 0.001 |
| Spi-B transcription factor (Spi-1/PU.1 related)[Source:HGNC Symbol;Acc:HGNC:11242] | 6.07E+02 | 1.47E+02 | 0.243 | 0.001 |
| dysbindin (dystrobrevin binding protein 1) domaincontaining 1 [Source:HGNC Symbol;Acc:HGNC:28455] | 2.19E+02 | 5.33E+01 | 0.244 | 0.006 |
| acetylcholinesterase (Yt blood group) [Source:HGNCSymbol;Acc:HGNC:108] | 1.72E+02 | 4.26E+01 | 0.248 | 0.010 |
| serpin peptidase inhibitor, clade G (C1 inhibitor),member 1 [Source:HGNC Symbol;Acc:HGNC:1228] | 1.53E+02 | 3.78E+01 | 0.248 | 0.012 |
| interferon-induced protein 44 [Source:HGNCSymbol;Acc:HGNC:16938] | 7.74E+01 | 1.94E+01 | 0.251 | 0.043 |
| kelch domain containing 7B [Source:HGNCSymbol;Acc:HGNC:25145] | 1.23E+02 | 3.10E+01 | 0.253 | 0.020 |
| suppressor of cytokine signaling 3 [Source:HGNCSymbol;Acc:HGNC:19391] | 6.62E+03 | 1.68E+03 | 0.254 | 0.001 |
| interferon-induced protein with tetratricopeptiderepeats 3 [Source:HGNC Symbol;Acc:HGNC:5411] | 1.26E+02 | 3.20E+01 | 0.254 | 0.019 |
| Rh blood group, CcEe antigens [Source:HGNCSymbol;Acc:HGNC:10008] | 3.65E+02 | 9.30E+01 | 0.255 | 0.004 |
| solute carrier family 46, member 2 [Source:HGNCSymbol;Acc:HGNC:16055] | 2.31E+02 | 5.91E+01 | 0.256 | 0.007 |
| serine peptidase inhibitor, Kunitz type 1[Source:HGNC Symbol;Acc:HGNC:11246] | 3.67E+02 | 9.40E+01 | 0.256 | 0.004 |
| G0/G1 switch 2 [Source:HGNC Symbol;Acc:HGNC:30229]" | 1.01E+04 | 2.61E+03 | 0.257 | 0.001 |
| cysteine-rich secretory protein LCCL domaincontaining 2 [Source:HGNC Symbol;Acc:HGNC:25248] | 4.89E+02 | 1.26E+02 | 0.258 | 0.003 |
| platelet factor 4 variant 1 [Source:HGNCSymbol;Acc:HGNC:8862] | 2.48E+02 | 6.40E+01 | 0.258 | 0.007 |
| serine/threonine kinase 32C [Source:HGNCSymbol;Acc:HGNC:21332] | 4.52E+02 | 1.19E+02 | 0.264 | 0.003 |
| tumor necrosis factor, alpha-induced protein 8-like2 [Source:HGNC Symbol;Acc:HGNC:26277] | 3.85E+02 | 1.02E+02 | 0.264 | 0.004 |
| 2'-5'-oligoadenylate synthetase 3, 100kDa[Source:HGNC Symbol;Acc:HGNC:8088] | 1.85E+03 | 4.91E+02 | 0.266 | 0.001 |
| CCAAT/enhancer binding protein (C/EBP), delta[Source:HGNC Symbol;Acc:HGNC:1835] | 3.50E+03 | 9.40E+02 | 0.268 | 0.001 |
| major histocompatibility complex, class I, C[Source:HGNC Symbol;Acc:HGNC:4933] | 2.91E+03 | 7.81E+02 | 0.269 | 0.001 |
| RUN domain containing 3A [Source:HGNCSymbol;Acc:HGNC:16984] | 3.81E+02 | 1.03E+02 | 0.270 | 0.005 |
| proteinase 3 [Source:HGNC Symbol;Acc:HGNC:9495]" | 3.61E+02 | 9.79E+01 | 0.271 | 0.005 |
| troponin T type 1 (skeletal, slow) [Source:HGNCSymbol;Acc:HGNC:11948] | 1.21E+02 | 3.30E+01 | 0.273 | 0.027 |
| lymphocyte antigen 6 complex, locus E [Source:HGNCSymbol;Acc:HGNC:6727] | 3.71E+02 | 1.02E+02 | 0.274 | 0.005 |
| shroom family member 1 [Source:HGNCSymbol;Acc:HGNC:24084] | 3.37E+02 | 9.30E+01 | 0.276 | 0.006 |
| kinesin light chain 3 [Source:HGNCSymbol;Acc:HGNC:20717] | 9.08E+01 | 2.52E+01 | 0.278 | 0.045 |
| major histocompatibility complex, class II, DR beta1 [Source:HGNC Symbol;Acc:HGNC:4948] | 1.70E+02 | 4.75E+01 | 0.279 | 0.017 |
| asialoglycoprotein receptor 1 [Source:HGNCSymbol;Acc:HGNC:742] | 7.67E+02 | 2.14E+02 | 0.279 | 0.003 |
| suppression of tumorigenicity 14 (colon carcinoma)[Source:HGNC Symbol;Acc:HGNC:11344] | 6.24E+02 | 1.74E+02 | 0.279 | 0.004 |
| T-cell leukemia/lymphoma 1A [Source:HGNCSymbol;Acc:HGNC:11648] | 4.96E+03 | 1.39E+03 | 0.281 | 0.002 |
| CDC42 effector protein (Rho GTPase binding) 1[Source:HGNC Symbol;Acc:HGNC:17014] | 1.83E+02 | 5.14E+01 | 0.281 | 0.016 |
| tumor necrosis factor (ligand) superfamily, member10 [Source:HGNC Symbol;Acc:HGNC:11925] | 4.60E+02 | 1.30E+02 | 0.282 | 0.005 |
| histone cluster 1, H3h [Source:HGNCSymbol;Acc:HGNC:4775] | 6.49E+02 | 1.83E+02 | 0.282 | 0.004 |
| heparan sulfate (glucosamine) 3-O-sulfotransferase1 [Source:HGNC Symbol;Acc:HGNC:5194] | 1.40E+02 | 3.97E+01 | 0.283 | 0.025 |
| **Genes** | **Con** | **GDM** | **GDM vs. Con** | |
| **Top 100 over-expressed** | **Mean** | **Mean** | **Ratio** | **p value** |
| major histocompatibility complex, class I, B[Source:HGNC Symbol;Acc:HGNC:4932] | 3.10E+00 | 3.25E+03 | 1051.182 | 0.000 |
| glucose-6-phosphate isomerase [Source:HGNCSymbol;Acc:HGNC:4458] | 1.03E+00 | 8.53E+01 | 82.667 | 0.000 |
| tumor necrosis factor receptor superfamily, member12A [Source:HGNC Symbol;Acc:HGNC:18152] | 2.99E+01 | 1.45E+03 | 48.622 | 0.000 |
| ICOS ligand [Source:UniProtKB/Swiss-Prot;Acc:O75144] | 3.10E+00 | 1.35E+02 | 43.525 | 0.000 |
| leukocyte immunoglobulin-like receptor, subfamily A(with TM domain), member 2 [Source:HGNCSymbol;Acc:HGNC:6603] | 1.03E+00 | 4.26E+01 | 41.333 | 0.003 |
| serpin peptidase inhibitor, clade A (alpha-1antiproteinase, antitrypsin), member 1 [Source:HGNCSymbol;Acc:HGNC:8941] | 9.29E+00 | 3.33E+02 | 35.906 | 0.000 |
| leucine rich repeat containing 37, member A2[Source:HGNC Symbol;Acc:HGNC:32404] | 1.03E+00 | 3.39E+01 | 32.879 | 0.010 |
| collagen, type I, alpha 1 [Source:HGNCSymbol;Acc:HGNC:2197] | 4.13E+00 | 1.24E+02 | 30.061 | 0.000 |
| nuclear receptor interacting protein 3 [Source:HGNCSymbol;Acc:HGNC:1167] | 5.16E+00 | 1.54E+02 | 29.873 | 0.000 |
| eukaryotic translation initiation factor 5A-like 1[Source:HGNC Symbol;Acc:HGNC:17419] | 1.03E+00 | 2.91E+01 | 28.182 | 0.020 |
| laminin, beta 1 [Source:HGNC Symbol;Acc:HGNC:6486]" | 1.03E+00 | 2.71E+01 | 26.303 | 0.026 |
| coiled-coil domain containing 3 [Source:HGNCSymbol;Acc:HGNC:23813] | 1.03E+00 | 2.71E+01 | 26.303 | 0.026 |
| laminin, beta 3 [Source:HGNC Symbol;Acc:HGNC:6490]" | 1.24E+01 | 3.14E+02 | 25.364 | 0.000 |
| l(3)mbt-like 4 (Drosophila) [Source:HGNCSymbol;Acc:HGNC:26677] | 1.03E+00 | 2.52E+01 | 24.424 | 0.035 |
| cytochrome P450, family 2, subfamily D, polypeptide6 [Source:HGNC Symbol;Acc:HGNC:2625] | 2.06E+00 | 4.65E+01 | 22.545 | 0.004 |
| major histocompatibility complex, class I, B[Source:HGNC Symbol;Acc:HGNC:4932] | 1.13E+02 | 2.41E+03 | 21.222 | 0.000 |
| pleckstrin homology-like domain, family A, member 1[Source:HGNC Symbol;Acc:HGNC:8933] | 5.16E+00 | 9.60E+01 | 18.600 | 0.000 |
| claudin 1 [Source:HGNC Symbol;Acc:HGNC:2032]" | 2.06E+00 | 3.78E+01 | 18.318 | 0.013 |
| threonine synthase-like 2 (S. cerevisiae)[Source:HGNC Symbol;Acc:HGNC:25602] | 5.16E+00 | 7.56E+01 | 14.655 | 0.001 |
| collagen, type I, alpha 2 [Source:HGNCSymbol;Acc:HGNC:2198] | 5.16E+00 | 6.40E+01 | 12.400 | 0.004 |
| leukocyte immunoglobulin-like receptor, subfamily B(with TM and ITIM domains), member 3 [Source:HGNCSymbol;Acc:HGNC:6607] | 1.24E+01 | 1.36E+02 | 10.960 | 0.000 |
| NGFI-A binding protein 2 (EGR1 binding protein 2)[Source:HGNC Symbol;Acc:HGNC:7627] | 2.15E+02 | 1.99E+03 | 9.267 | 0.000 |
| prune homolog 2 (Drosophila) [Source:HGNCSymbol;Acc:HGNC:25209] | 7.22E+00 | 6.59E+01 | 9.126 | 0.007 |
| epithelial membrane protein 1 [Source:HGNCSymbol;Acc:HGNC:3333] | 1.24E+01 | 1.11E+02 | 9.003 | 0.001 |
| solute carrier family 2 (facilitated glucosetransporter), member 14 [Source:HGNCSymbol;Acc:HGNC:18301] | 9.29E+00 | 7.95E+01 | 8.559 | 0.004 |
| amyloid beta (A4) precursor protein-binding, familyA, member 2 [Source:HGNC Symbol;Acc:HGNC:579] | 8.25E+00 | 6.88E+01 | 8.337 | 0.007 |
| golgin A6 family-like 9 [Source:HGNCSymbol;Acc:HGNC:37229] | 8.25E+00 | 6.69E+01 | 8.102 | 0.009 |
| DEAD (Asp-Glu-Ala-Asp) box polypeptide 39B[Source:HGNC Symbol;Acc:HGNC:13917] | 2.68E+01 | 1.99E+02 | 7.407 | 0.000 |
| melanoma cell adhesion molecule [Source:HGNCSymbol;Acc:HGNC:6934] | 1.55E+01 | 1.11E+02 | 7.202 | 0.003 |
| thrombospondin 1 [Source:HGNCSymbol;Acc:HGNC:11785] | 1.63E+03 | 1.15E+04 | 7.043 | 0.000 |
| dual specificity phosphatase 6 [Source:HGNCSymbol;Acc:HGNC:3072] | 4.30E+02 | 2.99E+03 | 6.947 | 0.000 |
| prostaglandin E synthase [Source:HGNCSymbol;Acc:HGNC:9599] | 1.13E+01 | 7.75E+01 | 6.832 | 0.009 |
| integrin, alpha D [Source:HGNCSymbol;Acc:HGNC:6146] | 2.68E+01 | 1.75E+02 | 6.540 | 0.001 |
| meningioma (disrupted in balanced translocation) 1[Source:HGNC Symbol;Acc:HGNC:7180] | 2.27E+01 | 1.48E+02 | 6.533 | 0.002 |
| dual specificity phosphatase 4 [Source:HGNCSymbol;Acc:HGNC:3070] | 2.17E+01 | 1.41E+02 | 6.486 | 0.002 |
| syntaxin 1A (brain) [Source:HGNCSymbol;Acc:HGNC:11433] | 1.00E+02 | 6.46E+02 | 6.450 | 0.000 |
| glutamate receptor, metabotropic 2 [Source:HGNCSymbol;Acc:HGNC:4594] | 1.13E+01 | 7.27E+01 | 6.405 | 0.013 |
| major histocompatibility complex, class I, B[Source:HGNC Symbol;Acc:HGNC:4932] | 3.93E+02 | 2.51E+03 | 6.396 | 0.000 |
| chemokine (C-C motif) receptor 4 [Source:HGNCSymbol;Acc:HGNC:1605] | 4.02E+01 | 2.53E+02 | 6.287 | 0.000 |
| interferon induced transmembrane protein 5[Source:HGNC Symbol;Acc:HGNC:16644] | 8.25E+00 | 5.14E+01 | 6.223 | 0.033 |
| sphingosine-1-phosphate receptor 2 [Source:HGNCSymbol;Acc:HGNC:3169] | 2.37E+01 | 1.45E+02 | 6.126 | 0.002 |
| interleukin 1 receptor-like 1 [Source:HGNCSymbol;Acc:HGNC:5998] | 9.29E+00 | 5.62E+01 | 6.054 | 0.028 |
| natural killer cell cytotoxicity receptor 3 ligand1 [Source:HGNC Symbol;Acc:HGNC:42400] | 8.25E+00 | 4.94E+01 | 5.989 | 0.038 |
| matrilin 1, cartilage matrix protein [Source:HGNCSymbol;Acc:HGNC:6907] | 1.55E+01 | 9.21E+01 | 5.949 | 0.009 |
| Wiskott-Aldrich syndrome-like [Source:HGNCSymbol;Acc:HGNC:12735] | 1.34E+01 | 7.85E+01 | 5.853 | 0.014 |
| FOS-like antigen 1 [Source:HGNCSymbol;Acc:HGNC:13718] | 1.11E+02 | 6.38E+02 | 5.723 | 0.000 |
| gamma-aminobutyric acid (GABA) A receptor, epsilon[Source:HGNC Symbol;Acc:HGNC:4085] | 1.24E+01 | 7.08E+01 | 5.715 | 0.019 |
| ryanodine receptor 3 [Source:HGNCSymbol;Acc:HGNC:10485] | 8.25E+00 | 4.65E+01 | 5.636 | 0.049 |
| free fatty acid receptor 1 [Source:HGNCSymbol;Acc:HGNC:4498] | 2.58E+01 | 1.44E+02 | 5.599 | 0.003 |
| nuclear receptor subfamily 4, group A, member 3[Source:HGNC Symbol;Acc:HGNC:7982] | 5.98E+01 | 3.34E+02 | 5.588 | 0.000 |
| matrix metallopeptidase 8 [Source:HGNCSymbol;Acc:HGNC:7175] | 4.23E+02 | 2.33E+03 | 5.499 | 0.000 |
| hes family bHLH transcription factor 1 [Source:HGNCSymbol;Acc:HGNC:5192] | 1.24E+01 | 6.78E+01 | 5.480 | 0.023 |
| ermin, ERM-like protein [Source:HGNCSymbol;Acc:HGNC:29208] | 2.06E+01 | 1.11E+02 | 5.402 | 0.008 |
| mitogen-activated protein kinase 6 [Source:HGNCSymbol;Acc:HGNC:6879] | 4.23E+01 | 2.27E+02 | 5.361 | 0.001 |
| NADH dehydrogenase (ubiquinone) flavoprotein 2,24kDa [Source:HGNC Symbol;Acc:HGNC:7717] | 1.24E+01 | 6.40E+01 | 5.167 | 0.031 |
| antigen p97 (melanoma associated) identified bymonoclonal antibodies 133.2 and 96.5 [Source:HGNCSymbol;Acc:HGNC:7037] | 1.86E+01 | 9.30E+01 | 5.010 | 0.015 |
| hemoglobin, zeta [Source:HGNCSymbol;Acc:HGNC:4835] | 1.96E+01 | 9.69E+01 | 4.944 | 0.014 |
| sphingosine-1-phosphate phosphatase 2 [Source:HGNCSymbol;Acc:HGNC:19953] | 2.27E+01 | 1.10E+02 | 4.868 | 0.011 |
| ureidopropionase, beta [Source:HGNCSymbol;Acc:HGNC:16297] | 1.34E+01 | 6.49E+01 | 4.841 | 0.036 |
| phosphodiesterase 4D, cAMP-specific [Source:HGNCSymbol;Acc:HGNC:8783] | 2.45E+02 | 1.18E+03 | 4.840 | 0.000 |
| haptoglobin [Source:HGNC Symbol;Acc:HGNC:5141]" | 5.02E+02 | 2.43E+03 | 4.830 | 0.000 |
| A kinase (PRKA) anchor protein 12 [Source:HGNCSymbol;Acc:HGNC:370] | 1.24E+01 | 5.91E+01 | 4.775 | 0.044 |
| FSHD region gene 1 family, member B [Source:HGNCSymbol;Acc:HGNC:15792] | 1.24E+01 | 5.91E+01 | 4.775 | 0.044 |
| orosomucoid 1 [Source:HGNC Symbol;Acc:HGNC:8498]" | 1.22E+02 | 5.69E+02 | 4.673 | 0.001 |
| zinc finger protein 462 [Source:HGNCSymbol;Acc:HGNC:21684] | 2.79E+01 | 1.30E+02 | 4.662 | 0.009 |
| ectodermal-neural cortex 1 (with BTB domain)[Source:HGNC Symbol;Acc:HGNC:3345] | 1.70E+02 | 7.77E+02 | 4.566 | 0.001 |
| hypermethylated in cancer 1 [Source:HGNCSymbol;Acc:HGNC:4909] | 9.49E+01 | 4.30E+02 | 4.534 | 0.001 |
| SMAD family member 7 [Source:HGNCSymbol;Acc:HGNC:6773] | 2.32E+02 | 1.05E+03 | 4.530 | 0.000 |
| ankyrin repeat domain 36 [Source:HGNCSymbol;Acc:HGNC:24079] | 1.44E+01 | 6.49E+01 | 4.496 | 0.043 |
| matrix metallopeptidase 9 [Source:HGNCSymbol;Acc:HGNC:7176] | 1.35E+02 | 5.99E+02 | 4.432 | 0.001 |
| kinesin family member 19 [Source:HGNCSymbol;Acc:HGNC:26735] | 1.55E+01 | 6.78E+01 | 4.384 | 0.042 |
| kinesin family member 16B [Source:HGNCSymbol;Acc:HGNC:15869] | 1.96E+01 | 8.43E+01 | 4.301 | 0.030 |
| lipocalin 2 [Source:HGNC Symbol;Acc:HGNC:6526]" | 1.83E+03 | 7.73E+03 | 4.234 | 0.000 |
| tumor necrosis factor receptor superfamily, member21 [Source:HGNC Symbol;Acc:HGNC:13469] | 2.06E+01 | 8.72E+01 | 4.227 | 0.030 |
| Iduronate 2-sulfatase (Hunter syndrome) [Source:UniProtKB/TrEMBL;Acc:B3KWA1] | 3.20E+01 | 1.35E+02 | 4.212 | 0.013 |
| zinc finger and BTB domain containing 43[Source:HGNC Symbol;Acc:HGNC:17908] | 1.83E+02 | 7.68E+02 | 4.203 | 0.001 |
| tumor protein p53 inducible nuclear protein 2[Source:HGNC Symbol;Acc:HGNC:16104] | 1.53E+02 | 6.39E+02 | 4.183 | 0.001 |
| cathelicidin antimicrobial peptide [Source:HGNCSymbol;Acc:HGNC:1472] | 2.62E+03 | 1.07E+04 | 4.070 | 0.000 |
| GRP1 (general receptor for phosphoinositides 1)-associated scaffold protein [Source:HGNCSymbol;Acc:HGNC:18707] | 1.34E+03 | 5.43E+03 | 4.067 | 0.001 |
| oxidized low density lipoprotein (lectin-like)receptor 1 [Source:HGNC Symbol;Acc:HGNC:8133] | 2.89E+01 | 1.17E+02 | 4.060 | 0.020 |
| transcobalamin I (vitamin B12 binding protein, Rbinder family) [Source:HGNC Symbol;Acc:HGNC:11652] | 7.84E+01 | 3.18E+02 | 4.054 | 0.004 |
| G protein-coupled receptor associated sortingprotein 1 [Source:HGNC Symbol;Acc:HGNC:24834] | 1.52E+02 | 6.11E+02 | 4.026 | 0.002 |
| coiled-coil domain containing 85C [Source:HGNCSymbol;Acc:HGNC:35459] | 2.47E+02 | 9.85E+02 | 3.993 | 0.001 |
| zinc finger protein 532 [Source:HGNCSymbol;Acc:HGNC:30940] | 4.95E+01 | 1.98E+02 | 3.992 | 0.008 |
| Fc fragment of IgG, low affinity IIIb, receptor(CD16b) [Source:HGNC Symbol;Acc:HGNC:3620] | 5.06E+01 | 1.95E+02 | 3.853 | 0.010 |
| Leucine zipper putative tumor suppressor 3[Source:UniProtKB/Swiss-Prot;Acc:O60299] | 3.61E+01 | 1.36E+02 | 3.758 | 0.021 |
| ring finger protein 207 [Source:HGNCSymbol;Acc:HGNC:32947] | 2.79E+01 | 1.04E+02 | 3.723 | 0.033 |
| Interferon regulatory factor 9[Source:UniProtKB/TrEMBL;Acc:A0A0B4J293] | 2.37E+01 | 8.82E+01 | 3.717 | 0.043 |
| regulator of G-protein signaling 1 [Source:HGNCSymbol;Acc:HGNC:9991] | 1.56E+02 | 5.77E+02 | 3.702 | 0.003 |
| regulator of G-protein signaling 16 [Source:HGNCSymbol;Acc:HGNC:9997] | 4.13E+01 | 1.52E+02 | 3.687 | 0.018 |
| siah E3 ubiquitin protein ligase 1 [Source:HGNCSymbol;Acc:HGNC:10857] | 3.40E+01 | 1.25E+02 | 3.672 | 0.026 |
| v-rel avian reticuloendotheliosis viral oncogenehomolog [Source:HGNC Symbol;Acc:HGNC:9954] | 1.12E+02 | 4.11E+02 | 3.654 | 0.005 |
| fem-1 homolog c (C. elegans) [Source:HGNCSymbol;Acc:HGNC:16933] | 4.95E+01 | 1.80E+02 | 3.640 | 0.015 |
| transmembrane protein 88 [Source:HGNCSymbol;Acc:HGNC:32371] | 7.33E+01 | 2.67E+02 | 3.638 | 0.009 |
| TCDD-inducible poly(ADP-ribose) polymerase[Source:HGNC Symbol;Acc:HGNC:23696] | 3.92E+01 | 1.42E+02 | 3.634 | 0.022 |
| peptidylprolyl isomerase F [Source:HGNCSymbol;Acc:HGNC:9259] | 1.66E+03 | 5.97E+03 | 3.585 | 0.001 |
| cytochrome P450, family 4, subfamily V, polypeptide2 [Source:HGNC Symbol;Acc:HGNC:23198] | 9.80E+01 | 3.50E+02 | 3.570 | 0.007 |
| histidine decarboxylase [Source:HGNCSymbol;Acc:HGNC:4855] | 1.80E+02 | 6.30E+02 | 3.509 | 0.004 |
| polo-like kinase 3 [Source:HGNCSymbol;Acc:HGNC:2154] | 1.06E+03 | 3.68E+03 | 3.481 | 0.002 |
| UDP-GlcNAc:betaGal beta-1,3-N-acetylglucosaminyltransferase 5 [Source:HGNCSymbol;Acc:HGNC:15684] | 3.71E+01 | 1.29E+02 | 3.471 | 0.030 |
